# Application of Bioelectrical Impedance Analysis to Detect Broiler Breast Filets Affected With Woody Breast Myopathy

**DOI:** 10.3389/fphys.2020.00808

**Published:** 2020-07-10

**Authors:** Amit Morey, Avery E. Smith, Laura Jewell Garner, Marlin K. Cox

**Affiliations:** ^1^Department of Poultry Science, Auburn University, Auburn, AL, United States; ^2^CQ Foods, Inc., Juneau, AK, United States

**Keywords:** woody breast, bioelectric impedance, resistance, reactance, broiler – chicken

## Abstract

Woody breast (WB) myopathy in modern broilers is causing major meat quality issues and consumer complaints. The poultry industry is sorting out WB filets through the inconsistent manual hand-palpation method. Bioelectrical impedance analysis (BIA) method was evaluated as a rapid and objective WB detection method. Freshly deboned broiler breast filets (15 filets × 2 categories × 3 trials) were sorted (hand-palpation) into severe woody (SW) and normal (N) categories were analyzed for BIA values, cook loss, texture (BMORS) method. SW filets had significantly (*P* < 0.05) higher resistance and reactance compared to N indicating BIA can be used to detect WB filets. In another experiment, we determined the ability of the BIA to differentiate between four WB severity levels using the whole filet. Significant differences were observed in resistance and reactance of normal and other WB categories, however, there were no significant differences among mild, moderate and severe WB categories. Segmental BIA of those filets indicated that BIA can be used to separate cranial, medial and caudal region of the breast filet based on the presence of WB myopathy. Accidental discovery of spaghetti breast in the samples demonstrated the significance of compounding different factors in analyzing WB meat using BIA.

## Introduction

Woody breast (WB) myopathy in modern broilers is causing major meat quality issues and consumer complaints and this problem is further exasperated by industry not having a reasonable objective means to detect it. Convenience, versatility, variety, and health benefits have increased the rate of consumed chicken in the United States from 36 lbs/capita/year in 1965 to approximately 94 lbs/capita/year in 2018 (National Chicken Council, 2020a). To satisfy the growing demand, the broiler industry has developed fast-growing big-broiler strains (>6 lbs live wt.) that culminated in over 42 billion lbs in 2018 of ready-to-cook poultry meat (National Chicken Council, 2020b).

The fast-growing broiler strains have developed a degenerative muscle myopathy in the pectoralis major termed as woody breast myopathy WB which was first reported by Dr. S. Bilgili at Auburn University in 2013 (Bilgili, 2013). Tijare et al. (2016) reported that the incidence of WB in United States broilers was 96.1%, with 48% exhibiting mild WB, 28% exhibiting moderate, and 20% exhibiting severe WB. Filets with WB are hard (very dense) to the touch with varying degrees of hardness due to collagen infiltration along the ventral area of the breast. WB condition is difficult to detect because it can vary in the degree of hardness, be focal or diffuse within the breast and, be randomly distributed within a broiler flock (Tijare et al., 2016). Histology of WB indicates muscle fiber fragmentation, hyalinization, swelling of myofibers, necrotic muscle fiber replacement with connective tissue (fibrosis), macrophage infiltration, and presence of irregular patches of adipose tissue (Bilgili, 2013; Velleman and Clark, 2015). Collagen infiltration is a major causes of WB hard texture and the infiltration pattern differs among broiler strains (Sihvo et al., 2014; Velleman and Clark, 2015; Tijare et al., 2016). Biochemical analysis of filets indicates higher moisture (4.5%) lower protein (4%) content than normal filets (Wold et al., 2017).

Woody breast is apparent to average consumers as well as to culinary experts. Breasts exhibiting WB characteristics have a hard texture and are both visually and texturally unappealing leading to low overall consumer acceptance (Petracci et al., 2015). Filets affected with WB have approximately 50% lower marinade uptake and approximately 27% higher cook loss compared to normal breast meat (Mudalal et al., 2015). As a result, poultry processors have to either deal with rejected orders and complaints or if WB is identified prior to sale, offer the product as lower quality product for a reduced price and absorb significant profit losses (Mudalal et al., 2015; Petracci et al., 2015).

Woody breast myopathy is a meat quality issue faced by the global poultry industry. Although established and identified in the United States, WB is now being detected in other countries including the Italy, Denmark, United Kingdom, and Finland (Sihvo et al., 2014; Trocino et al., 2015; Brot et al., 2016; Larsen et al., 2016; Tijare et al., 2016). A study from Italy (Trocino et al., 2015) observed that males from two broiler genotypes had 3-times higher occurrence of WB than females.

Detection methods used by industry are primarily hand palpation which are subjective, problematic, and unreliable. Typically, a plant will train employees to hand palpate the breast filets as they are passing on a conveyor to sense filet hardness and subsequently classify them into ranked severities on processor specific thresholds making industry wide protocols non-existent (Table 1). Personal observations and discussions with United States poultry processors indicate that hand-palpation is a subjective evaluation method, can give false-positive/negative WB scores, pass-on WB to customers, and remove high-valued normal filets thus affecting the profits and quality. Hand-palpation is also costly since 8–10 additional trained personnel will have to be employed per line to sort WB filets. A standard objective measure is needed for industry. Other technologies are available that can provide data rich objective measures of products at the cellular level and these may bridge the gap needed for industry to utilize an industry wide objective measure. Recently, Wold et al. (2017) reported the successful application of near infrared spectroscopy as an on-line method to detect WB in processing plants.

**TABLE 1:** Various scales used to categorize woody breast.

2-point scale	3-point scale	4-point scale Tijare et al., 2016
0 – Normal (no woody breast or toughness)	0 – Normal (no woody breast or toughness)	0 – Normal (filets flexible throughout)
1 – Woody breast (tough filet)	1 – Medium woody (upto 50% woody)	1 – Mild (filets hard mainly in the cranial region but flexible otherwise)
	2 – Severe woody (>50% woody)	2 – Moderate (filets extremely hard and rigid through from cranial region of caudal tip filets that were hard throughout but flexible in mid-to caudal region)
		3 – Severe (>50% of filet area is woody)

Bioelectrical Impedance Analysis (BIA) is a technology that has been proven to measure many different properties at the cellular level and has been used extensively on a number of organisms ranging from fish to humans and may provide a solution for WB detection. It provides instant results, is easy to use and objective. BIA measures the electrical properties of both living and harvested plants and animals. Electrical properties measured are resistance and reactance which dependend on changes in cell membrane/wall integrity and extra- and intracellular fluids (Kyle et al., 2004). Although initially designed as a human medical device, BIA has been used to extensively to non-invasively measure proximate composition, health, and freshness of fish and meats (Swatland, 2002; Cox and Hartman, 2005; Chevalier et al., 2006). A new application of BIA may be to detect WB myopathy. The objective of this study was to conduct a proof-of-concept research to evaluate if WB leads to alteration in bioelectrical properties such that BIA can be used to detect filets. Since processors differentiate breast filets based on varying severity levels of WB myopathy, experiments were conducted to determine if BIA could differentiate between those severity levels. It would also be beneficial for the processors to detect which segment of the breast filet has WB so that it can be excised, and the rest of the filet sold at a higher price. Segmental BIA analysis of the cranial, medial and caudal segments of the intact filets was conducted to determine if BIA can differentiate between normal and WB categories.

## Materials and Methods

### Experiment 1

#### Proof-of-Concept

Freshly deboned breast filets were sorted into normal and severe WB meat and analyzed for bioelectrical impedance (resistance and reactance), cook loss, and texture.

### Broiler Breast Meat

Freshly deboned (2–3 h post-mortem) butterfly breast filets from 8-wk broilers (all male; Ross 708; and 8–9 lbs live weight) were obtained from a local broiler processor. All samples were transported to the Department of Poultry Science, Auburn University under refrigeration and analyzed (except texture) within 2–3 h. The left filet from each the butterfly filet was cut and were immediately separated into normal (no WB) and severe WB using hand-palpation method (Tijare et al., 2016).

### Bioelectrical Impedance Analysis

**FIGURE 1:**
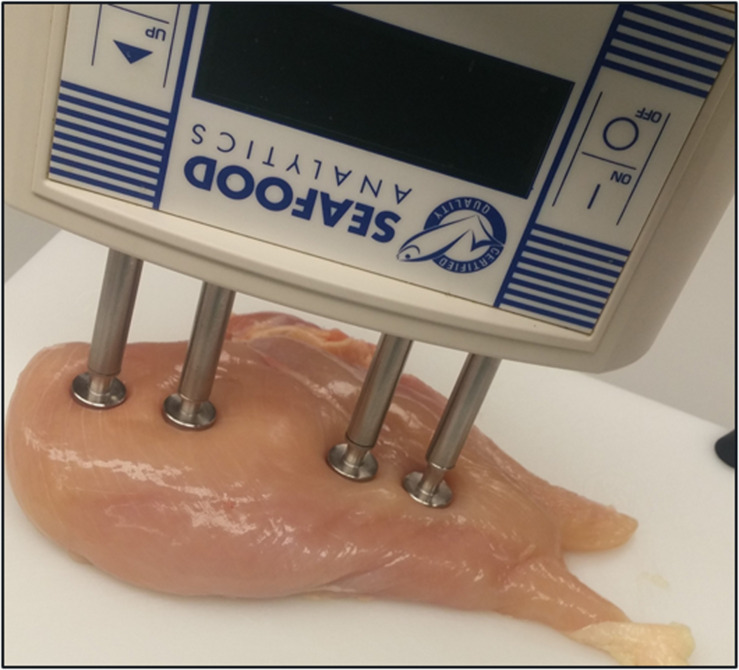
Bioelectrical impedance analysis of whole breast filet.

Left-side filet was used to measure bioelectric properties using the hand-held BIA equipment (Seafood Analytics, Clinton Town, MI, United States) on the dorsal side of the filet (Figure 1). The BIA unit consists of two signal electrodes and two detecting electrodes that introduce an 800 μA, 50 kHz, AC current capable of voltage changes between 3.75–10.60 V. The four electrodes are connected to the product using food grade stainless compression electrodes (RJL Systems, Detroit, MI, United States). A four-electrode array is used to approximate parallel field lines within the tissue, negate product electrode interfaces, and approximate a cylindrical shape. Once the electrodes are in contact with the product, the circuit is connected, and the device takes two measures, resistance and reactance. Data was collected on the dorsal surface (feather side) of the filets weighing 464 ± 66 *g*.

### Cook Loss

Left-side filet used for BIA measurement was further used for cook loss analysis immediately (<15 min) after BIA measurement (approximately 5 h post-mortem). Cook loss is expressed as weight loss after cooking the thigh relative to its initial weight. Briefly, individual filet were weighed, placed on a raised stainless steel wire rack in a stainless-steel pan (53.02 × 32.54 × 10.16 cm; Vollrath Co., LLC, Sheboygan, WI, United States), covered with aluminum foil and cooked in a pre-heated (176.6°C) forced air convection oven (Vulcan HEC5D, Troy, OH, United States) to an internal temperature of 74°C measured using a stainless-steel digital thermometer (Taylor 1470FS Digital cooking thermometer and Kitchen Timer, Las Cruces, NM, United States). After cooking, the filets were cooled to room temperature (22 ± 2°C) in the covered pans and then reweighed. Cook loss was calculated using the following formula:

$$\begin{array}{c} \mathrm{Cook loss}\;(\%)\\ \;=100\times \frac{\left(\mathrm{Initial weight of thigh}-\mathrm{cooked weight of thigh}\right)}{\left(\mathrm{Initial weight of thigh}\right)} \end{array}$$

(Kennedy-Smith et al., 2017).

### Texture Analysis

The cooked filet used for cook loss analysis was placed individually in separate zip-loc bags and stored overnight at 4°C. The filets were tempered at room temperature for 3–4 h and analyzed for texture using the Blunt Mullenet-Owens Razor Shear (BMORS) method Texture Analyzer (Model TX-XT2i, Technologies, Scarsdale, NY, United States; Lee et al., 2008; Morey and Johnson, 2019).

### Statistical Analysis

Breast filets (15 filets/severity/trial) belonging to normal and severe WB category were obtained and the experiment was conducted in 3 separate replicate trials (15 filets × 2 severity levels × 3 replicate trials = 90 filets) were used throughout the study. Replications were tested using one-way ANOVA with Tukeys HSD (*p* < 0.05). to determine significant differences between replications. In the absence of significant differences in replications, the data from all replications was combined and analyzed together. Data was analyzed one-way ANOVA with Tukeys HSD to determine significant differences at *p* < 0.05.

### Experiment 2

#### Segmental Bioelectrical Impedance Analysis

**FIGURE 2:**
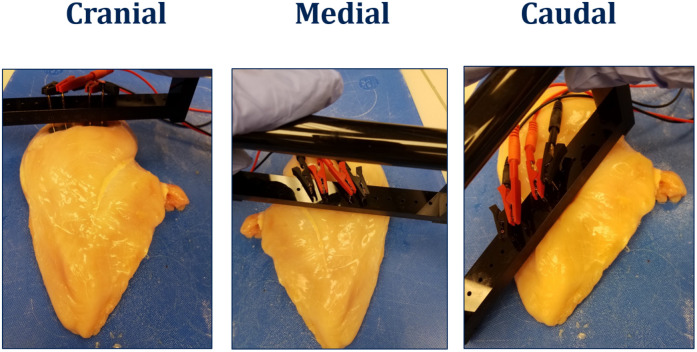
Segmental BIA of breast filets.

Freshly deboned chicken breast filets were randomly collected (without sorting into WB categories; all male; Ross 708; 8–9 lbs live weight) from the same poultry processor. The filets were stored for 18 h at 4°C prior to the analysis. Each filet was sorted into one of the WB categories (0, 1, 2, and 3) and the cranial segment was pinched to evaluate the turgor to determine the presence/absence of spaghetti meat. The filet was then analyzed for bioelectrical impedance (resistance and reactance) as per the method above. Each filet was visually divided into three segments, cranial, medial, and caudal region and hand-palpated to determine if the segment was normal (no perceivable hardness) and woody (perceived hardness). Each segment was then analyzed for bioelectrical impedance (resistance and reactance) using two sets of small (5.08 cm) compression electrodes with 1 cm between signaling and receiving electrode (Figure 2).

### Statistical Analysis

Freshly processed 120 random filets were collected on three separate processing days and analyzed for bioelectrical impedance. Data was combined from all processing days and analyzed for differences in resistance and reactance of different WB categories of the whole filet and the segments with and without spaghetti meat using one-way ANOVA with Tukey’s HSD to separate means at *p* < 0.05. The segmental BIA data (resistance and reactance) on the cranial and caudal region with and without spaghetti meat was analyzed using linear discriminant analysis (LDA) with 60% of the data used for training and 40% was used for validation. The prediction accuracy (%) and error (%) of the model to differentiate each segment into normal and woody was determined (Wold et al., 2017).

## Results

### Experiment 1

The differences in the quality characteristics and the bioelectrical impedance parameters is given in Table 2. Breast filets affected with severe woody (SW) breast myopathy WB have a significantly higher (*p* ≤ 0.05) cook loss (41.17%) compared to normal breast filets (35.16%) indicating that WB leads to reduced water holding capacity of the meat (Table 2). BMORS texture analysis indicated that WB meat had significantly higher (*p* ≤ 0.05) peak counts (10.77) compared to normal breast meat (5.45). However, the peak shear force and total energy for normal meat were higher (*p* ≤ 0.05) than severe WB meat. Bioelectrical properties (resistance and reactance) were measured using the hand-held CQ Reader (Table 2). Overall, data indicates that there was a significant difference (*p* ≤ 0.05) in resistance (Normal: 72.18 Ω vs Woody: 78.27 Ω), and reactance (Normal: 28.04 Ω vs Woody: 37.54 Ω) of breast meat affected by WB myopathy.

**TABLE 2:** Differences in cook loss, texture (peak count, shear force, and shear energy) and BIA values (resistance and reactance) of normal and severe woody breast filets (mean ± std dev).

Variable	Normal (*n* = 45)	Severe woody (*n* = 45)	*p*-value
Cook loss (%)	35.16 ± 3.09	41.17 ± 4.36	<0.0001
Peak count	5.45 ± 2.32	10.77 ± 2.33	<0.0001
Peak shear force (*N*)	21.66 ± 3.43	18.17 ± 4.90	0.0004
Total energy (*N*.mm)	277.47 ± 39.50	242.07 ± 64.77	0.0043
Resistance (*R*; Ω)	72.18 ± 12.86	78.27 ± 11.14	0.028
Reactance (*X*_c_; Ω)	28.04 ± 10.22	37.54 ± 9.86	<0.0001

### Experiment 2

Freshly procured breast filets were hand-palpated to separate them into varying WB categories (Tijare et al., 2016; Table 1) and detected for the presence of spaghetti meat. Out of the total 360 filets analyzed, there were 15.56% normal, 22.5% mild, 35.56% moderate, and 35.56% severe (Table 3). The resistance and reactance values were overall significantly different. Normal and WB meat had significantly different resistance (74.19 vs 69.43, respectively), and reactance (19.58 vs 23.49 Ω, respectively; Table 3). However, there were not significant differences (*p* > 0.05) in the resistance between mild, moderate and severe WB. No significant differences in reactance were observed between normal, mild and moderate WB (Table 3). Similar data trends in resistance and reactance were observed when the data of whole filets without spaghetti meat was removed (Table 3). A total of 73 filets out of 360 had spaghetti breast myopathy.

**TABLE 3:** BIA analysis of whole breast filets analyzed with and without the filets containing spaghetti meat.

	Whole filets with spaghetti meat			Whole filets without spaghetti meat		
Woody breast score	*n*	Resistance	Reactance	*n*	Resistance	Reactance
Normal	55	74.19^A^	19.58^B^	31	70.71^A^	19.10^B^
Mild	80	66.09^B^	17.62^B^	50	65.67^B^	18.11^B^
Moderate	93	66.37^B^	20.60^AB^	79	66.18^B^	20.59^B^
Severe	129	69.43^B^	23.49^A^	124	66.12^AB^	23.36^A^
*p*-value		0.0002	<0.0001		0.0013	<0.0001
CV (%)*		13.38	32.04		13.69	31.94

Superscripts ^A–B^ indicate significant differences (p < 0.05) between woody breast scores in each column. *Coefficient of Variation (%).

Overall, the normal and WB occurrence in the cranial region was 27.78 and 72.22%, respectively, medial region 40.56 and 31.67%, respectively, and caudal regions was 50 and 50%, respectively. Spaghetti breast was prevalent mostly (24–32%) in the normal segments while its occurrence was lower in the woody segment (10–18%). Contrary to the whole filet resistance and reactance, the normal segments had a lower resistance compared to the woody segments. Resistance and reactance of the cranial and medial segments, respectively, were significantly different while they were not significantly different for the caudal region. Removal of the resistance and reactance data from spaghetti meat segments did not alter the differences in normal and WB meat (Table 4). However, LDA of the cranial segment data using resistance and reactance could accurately predict normal and woody segments at 68.69 and 57.75%, respectively. However, when the spaghetti meat data was removed, the prediction accuracy of both normal and woody segments increased by approx. 3%. When resistance and reactance data from the cranial segment affected by spaghetti meat was analyzed, the model was able to predict normal and WB at 52–54% accuracy (Table 5).

**TABLE 4:** BIA Analysis of breast filet segments analyzed with and without filets containing spaghetti meat.

Breast filet segment	Woody breast presence	Filet segments with spaghetti meat			Filet segments without spaghetti meat		
		*n*	Resistance (Ω)	Reactance (Ω)	*n*	Resistance (Ω)	Reactance (Ω)
Cranial	Normal	100	74.96^B^	18.24^B^	56	74.76^B^	17.47^B^
	Woody	260	84.20^A^	21.42^A^	228	85.95^A^	22.20^A^
	*p*-value		<0.0001	0.0047		<0.0001	0.0013
	CV (%)*		17.71	45.88		17.10	45.58
Medial	N	146	67.47^B^	15.63^B^	97	67.37^B^	15.71^B^
	W	114	76.82^A^	18.33^A^	86	78.06^A^	18.34^A^
	*p*-value		<0.0001	0.0013		<0.0001	0.0075
	CV (%)*		17.33	44.98		17.34	44.74
Caudal	N	180	80.13^A^	18.40^A^	122	80.85^A^	18.26^A^
	W	180	81.38^A^	18.60^A^	161	83.00^A^	18.78^A^
	*p*-value		0.4148	0.8345		0.2167	0.6227
	CV (%)*		18.00	47.62		17.55	46.74

Superscripts ^A–B^ indicate significant differences (p < 0.05) between normal and woody breast for each segment (cranial, medial, and caudal). *Coefficient of Variation (%).

**TABLE 5:** Discriminate analysis of woody and normal classification in filet segments to determine accuracy and error.

	Normal		Woody	
	% Accuracy	% Error	% Accuracy	% Error
Cranial segment (all filets)	68.69	31.31	57.75	42.25
Cranial segment (no spaghetti)	72.73	27.27	61.33	38.67
Cranial segment (only spaghetti)	52.27	47.73	54.55	45.45
Medial segment (all data)	70.55	29.45	54.42	45.58
Medial segment (no spaghetti)	73.53	26.47	59.59	50.44
Medial segment (only spaghetti)	57.14	42.86	11.11	88.89

## Discussion

### Experiment 1

Woody breast myopathy is a major meat quality issue in the poultry industry. At the macro level, raw WB meat is hard to touch while Aguirre et al. (2018) reported that taste panelists described cooked WB meat crunchy and fibrous. At the molecular level, WB is characterized with the conformational changes in muscle protein lead to higher extra-myofibrillar water in WB compared to normal meat (Tasoniero et al., 2017). Previous research conducted in our lab using 7–T Magnetic Resonance Imaging of woody and normal breast filets showed that the WB filets had had significantly higher interstitial water compared to normal filets (Kennedy-Smith et al., 2017) which potentially alters the bioelectrical properties of breast filets.

The differences in the intra- and extra-cellular water can impact meat quality parameters such as cook loss. Higher cook loss in WB filets may also indicate higher levels of free water that can be easily removed from meats due to cooking. Higher levels of free water can be a result of increased accumulation of collagen in the WB filets preventing the binding of free water to the myofibrillar proteins (Kennedy-Smith et al., 2017). Textural differences in WB (Table 2) can be attributed to the alternation in muscle architecture. Histological analysis of WB indicates fibrosis, perimysial thickening, proliferation of connective tissue and a higher collagen content (Soglia et al., 2016) which can potentially contribute to differences in texture of the WB meat. Similar to the current study Solo (2016) also observed that the total shear energy increased as the severity of the filets increased from normal to severe WB. Moreover, the author stated that the normal filets had a peak count of 5.73 which was significantly lower than severe WBs (8.79) which was similar to the current research. Peak count measurements obtained through BMORS method can distinguish between normal and WB filets.

Cook loss and texture analysis are destructive methods to differentiate between normal and WB. Moreover, these methods are laboratory intensive and are not suitable for in-plant application. Hence the hand-held BIA equipment was used to determine if there are bioelectrical differences between normal and WB meat such that those difference can be further used to detect WB from normal breast meat.

Resistance (*R*_s_) measures the ability of a substance to conduct electricity while reactance (*X*_c_) measures its ability to hold a charge (Lukaski, 1987) which are influenced by the biochemical composition of food (Kyle et al., 2004; Hafs and Hartman, 2011). As observed in the cook loss data (Table 2), the changes in the WB muscle architecture can influence the disposition of water within the tissue which can result in alteration in the electrical properties of the meat. It was observed that normal breast filets had lower Rs and Xc (72.18 Ω and 28.04 Ω, respectively) compared to severe WB filets (78.27 Ω and 37.54 Ω, respectively). The resistance is impacted by intra- and extracellular water while reactance mainly arises from cell membranes (Kyle et al., 2004). Cells do not conduct electricity at low frequencies and hence act as insulators forcing the current to pass through the extracellular fluid, which was higher in severe WB (see section “Cook Loss” Table 2), thus increasing the resistance of the muscle (Kyle et al., 2004). Alternatively, Kyle et al. (2004) also state that increase in the suspended non-conducting material will increase the resistance of the conducting water. In case of WB breast myopathy, the non-conducting material could be connective tissue infiltration and granulation tissue (Sihvo et al., 2014; Velleman, 2015; Soglia et al., 2016) which increases the resistance of the meat. These changes in the muscle combined with differences in intra- and extra-cellular water influence the differences in the resistance and reactance of WB compared to the normal meat.

The proof-of-concept research indicates that BIA has the potential to be used as an effective tool to detect severe WB filets at a processing plant.

### Experiment 2

Tijare et al. (2016) noted that based on the severity, WB can be classified into normal, mild, moderate and severe (Table 2). Poultry processors have favored the 4-tier classification as they can remediate their losses by staggering the price or the utilization of breast meat depending on the severity level. It would be further beneficial for the poultry processors to determine which section of the breast filet has woody characteristics so that they can salvage the remaining breast filet and sell it at a higher cost. Since hand-palpation is very laborious and subjective, and that based on Experiment 1, we had demonstrated that BIA can be used to differentiate between severe and normal WB, a study was conducted to determine if BIA can be used to detect varying WB severities as well as segments of the breast filet that are affected by WB.

When compared to experiment 1, the normal breast filet in experiment 2 had similar resistance values, however, WB resistance reduced to approx. 69 Ω. This difference could be explained by the differences in the experimental setup. Compared to experiment 1 where the filets were analyzed within 6-h post-slaughter, in experiment 2 the filets were stored in the refrigerator for 18 h. Given the fact that the WB filets have higher extracellular water and modified muscle architecture, it exhibits higher drip loss during the first 24 h compared to the normal meat (Mudalal et al., 2015; Tasoniero et al., 2017; Sun et al., 2018) resulting in lower resistance in the stored WB meat in experiment 2. Significantly higher resistance of normal breast filet, similar to experiment 1, indicates that normal meat had a higher water holding capacity even after storage compared to WB. Higher reactance in WB meat can be attributed to the connective tissue and fibrosis, acting as insulators (Kyle et al., 2004). Accidental findings during the project indicated that presence of spaghetti breast myopathy (loose muscle fibers) can influence the resistance of the meat indicating that the lose muscle fibers in spaghetti meat act as insulators thus increasing the resistance values. The overlap in the varying severity levels can be explained by the design of the CQ Reader which has 2-sets of electrodes at set distances wherein each set takes replicate readings which are averaged and then presented as the data for the whole filet. However, with the varying severity WB levels, different regions in the breast filet may or may not have woody tissue and averaging the data for the entire filet can mask the effect of the differences in electrical properties of those regions. BIA measurement of segments of breast filet can provide a clearer picture of the presence of WB in each segment. The other major reason for the overlap could also be the difficulty in accurately detecting WB severities by hand-palpation especially after 18 h post-slaughter storage which impacts texture and inter- and extra-cellular water of the meat (Sun et al., 2018).

Segmental BIA of the cranial segment had the lowest percentage (27.78%) of normal breast followed by caudal (50%) and medial segments (40%). This shows that if the woody segment in the cranial region is accurately detected, the processor can remove that segment and still utilize the remaining breast filet. Significant differences were observed between normal and woody segments in terms of resistance and reactance. Contrary to the whole breast filet BIA, segmental BIA indicates higher resistance for woody than normal while the reactance pattern remains the same. Segmental BIA measures electrical properties of a localized area and had higher reactance indicating increased non-conducting material (fibers, connective, and granular tissue; Sihvo et al., 2014; Velleman, 2015; Soglia et al., 2016) in the water which would have resulted in higher resistance.

Discriminant analysis conducted using resistance and reactance values indicated that BIA can be used to differentiate between the normal and woody – cranial and medial segments. Although the resistance and reactance data with and without spaghetti were similar for each segment, removal of data of the spaghetti meat affected cranial and medial segments increased the prediction accuracy of normal and woody meat by approximately 3% (Table 5). Accidental finding of spaghetti breast meat and its electrical properties could be of high interest to the poultry industry. Significant differences in the BIA properties of breast meat with and without spaghetti meat indicate that the new myopathy should be taken into consideration while developing predictive models for WB myopathy using bioelectrical properties.

Bioelectrical impedance analysis can be used as a tool to differentiate between normal and severe breast meat. However, additional efforts are needed to further define and increase the accuracy of BIA to differentiate between varying severity levels. Increased sample number can potentially improve the discrimination ability of the model. Segmental BIA could be used as a tool to detect woody segments in the filets thus providing more granular data as well as better understanding of the spread of the myopathy in the muscle. The accidental finding on the interference of spaghetti breast meat in detecting WB can open new research areas to explore the ability of BIA to detect spaghetti breast myopathy. The research also demonstrates that BIA can alter with the freshness of the filets and each processor must develop the resistance and reactance threshold values based on their process. In its current state, the hand-held device can be used as a near-line technology by quality assurance departments to detect WB prevalence. Moreover, the data obtained by the processors can be used to study WB prevalence between flocks, different nutrition regime, as well as management practices. Further processors can use BIA technology to separate WB meat from the normal meat. Overall, the hand-held BIA technology can help in reducing consumer compliants due to WB.

## Data Availability Statement

The datasets generated for this study are available on request to the corresponding author.

## Author Contributions

AM is the lead PI who conceptualized the idea of using bioelectrical impedance analysis to detect woody breast, secured funding, and actively conducted the research. AS conducted the proof-of-concept experiment (Experiment 1) mentioned in the manuscript. LG conducted experiments, collected data and analyzed it. MC is the co-developer of the patented CQ Reader used for BIA experiments throughout the project, and supported with data analysis, technical knowledge, and writing manuscript. All authors contributed to the article and approved the submitted version.

## Conflict of Interest

MC was employed by the company CQ Foods. The remaining authors declare that the research was conducted in the absence of any commercial or financial relationships that could be construed as a potential conflict of interest.
